# Introducing a standardized assessment of patients’ interest in and usage of CAM in routine cancer care: chances and risks from patients’ and physicians’ point of view

**DOI:** 10.1007/s00432-023-05182-3

**Published:** 2023-09-16

**Authors:** M. Shalgouny, J. Bertz-Lepel, L. Fischer v. Weikersthal, J. Herbin, M. Meier-Höfig, R. Mücke, U. Rohe, T. Stauch, C. Stoll, D. Troeltzsch, S. Wittmann, O. Kurz, R. Naumann, J. Huebner

**Affiliations:** 1 Klinik für Innere Medizin II, Universitätsklinikum Jena, Am Klinikum 1, 07747 Jena, Germany; 2 Klinik für Hämatologie, Helios Klinikum Bad Saarow, Bad Saarow, Germany; 3 Praxis für Hämatologie und Internistische Onkologie, Gesundheitszentrum St. Marien GmbH, Amberg, Germany; 4 Onkologische Schwerpunktpraxis Berlin, Berlin, Germany; 5 3. Medizinische Klinik, Städtisches Krankenhaus Kiel, Kiel, Germany; 6 MVZ Strahlentherapie RheinMainNahe GmbH, Mainz, Germany; 7 St. Barbara Klinik Hamm, Hamm, Germany; 8 Klinik für Onkologie, Median Adelsbergklinik Bad Berka, Bad Berka, Germany; 9 Rehaklinik für Orthopädie, Klinik Herzoghöhe Bayreuth, Bayreuth, Germany; 10 Klinik für Mund-, Kiefer- und Gesichtschirurgie, Charité-Universitätsmedizin Berlin, Berlin, Germany; 11 Klinik für Onkologie, Hämatologie und Palliativmedizin, Helios Dr. Horst Schmidt Kliniken Wiesbaden, Wiesbaden, Germany; 12 Medizinische Klinik III, Marien Kliniken Siegen, Siegen, Germany

**Keywords:** Neoplasm, Complementary medicine, Interactions, Patient-physician communication, Cancer

## Abstract

**Background:**

Cancer patients often use complementary and alternative medicine (CAM), however, standardized assessment in clinical routine is missing. The aim of this study was to evaluate a screening questionnaire on CAM usage that was published in the S3 Guideline Complementary Medicine in the Treatment of Oncological Patients.

**Methods:**

We developed a survey questionnaire to assess the practicability of the guideline questionnaire and communication on CAM between health care providers (HCPs) and patients. We collected 258 guideline questionnaires and 116 survey questionnaires from ten clinics and held twelve semi-structured interviews with HCPs.

**Results:**

85% used at least one of the listed CAM methods, 54 participants (*N* = 77) never disclosed usage to a physician. The most frequently used CAM methods were physical activity (76.4%) and vitamin D (46.4%). 25.2% used at least one method, that was labeled risky by the guideline. 53.4% did not know of CAM’s risk of interactions and side effects. Introducing the guideline questionnaire in routine cancer care increased the rate of patients talking to an HCP regarding CAM significantly from 35.5 to 87.3%. The HCPs stated positive effects as an initiation of conversation, increased safety within CAM usage and patients feeling thankful and taken seriously. However, due to the limited amount of time available for discussions on CAM, generalized distribution to all patients was not feasible.

**Conclusion:**

Institutions should focus on implementing standard procedures and resources that help HCPs discuss CAM on a regular basis. HCPs should meet the patient’s demands for CAM counseling and make sure they are equipped professionally.

## Introduction

Over the past years, the use of complementary and alternative medicine (CAM) noticeably increased. The National Center for Complementary and Integrative Health defines the term CAM as a variety of methods such as nutritional supplements, mind–body-practices, and physical therapy (The National Center for Complementary and Integrative Health 2021). In the field of oncology, CAM developed a significant role as around 50% of cancer patients use some kind of CAM (Wortmann et al. 2016). Possible reasons for usage are to strengthen the immune system, become more active, prevent/treat side effects of anticancer treatments and increase the overall well-being (Wortmann et al. 2016; Gras et al. 2019). Cancer patients generally view CAM as a safe, holistic, natural, and nontoxic way to support their recovery (Davis et al. 2012). However, evidence about CAM is still insufficient and while undergoing cancer treatment unknown side effects and adverse interactions can occur (Conrad et al. 2014; Cramer et al. 2013). For this reason, it’s vital for cancer care providers to know of any complementary supplements that are taken by their patients. Still, many patients do not inform their oncologist or any physician about using CAM (Cramer et al. 2013; Wortmann et al. 2016). Reasons for nondisclosure are the doctor’s non inquiry, patient’s anticipation of doctor’s disapproval, disinterest, or inability to help, and the perception that the CAM use is irrelevant to their conventional care. There is a high probability that patient-physician communication about CAM use is associated with an enhanced patient-physician relationship and higher patient satisfaction (Davis et al. 2012; Stie et al. 2020). Improving communication regarding CAM ensures more disclosure, a safer usage as patient’s receive evidence-based information, and enables positive health outcomes with higher patient satisfaction (Akeeb et al. 2022).

In many healthcare providers (HCPs) acceptance of CAM is high. The likelihood of already being asked about CAM by cancer patients or their relatives is very high (Conrad et al. 2014). Many cancer care providers (CCPs) feel CAM options to be very important for treating disease-related as well as therapy-related symptoms (Klein and Guethlin 2018). But less than one-third think themselves adequately informed (Conrad et al. 2014; Trimborn et al. 2013). This lack of knowledge constitutes a barrier to open dialogue about CAM use with patients (Klein and Guethlin 2018).

A way to approach the existing lack of knowledge are systematically developed guidelines. Guidelines contribute to an improved quality of medical care. They provide recommendations to physicians and patients on appropriate health care for specific problems. The methodological characteristics in the development of guidelines are defined by stages. Guidelines of the third stage (S3) have the highest level of quality. These guidelines are developed by a full formalized, systematic guideline development process (Beyer et al. 2010). The German Program for Guidelines in Oncology has set the goal to develop and implement only the highest quality clinical practice guidelines in oncology, being S3 guidelines (Deutsche Krebsgesellschaft e.V. n.d.).

In the S3 Guideline Complementary Medicine in the Treatment of Oncological Patients the most important CAM methods in Germany are evaluated by evidence-based criteria. The aim of this guideline is to optimize the care of cancer patients. It is supposed to work as a reference, that provides physicians, other qualified personnel as well as patients evidence-based recommendations, that were processed by formal consensus. In addition to the guideline, a questionnaire for patients was developed, that lists all covered CAM methods. It was designed as a tool to reduce risks of CAM usage, especially interactions and side effects, given that HCPs apply the questionnaire to their work. The S3 guideline and the questionnaire should be a means to provide every cancer patient at every cancer care facility in Germany evidence-based answers to questions regarding CAM. If CCPs consider the recommendations of the guideline it could lead to better supportive care, strengthening of patient autonomy and adherence, and protection from side effects and interactions (Leitlinienprogramm Onkologie Deutsche Krebsgesellschaft 2021).

In this study, we trialed the questionnaire of the S3 guideline Complementary Medicine in the Treatment of Oncological Patients to evaluate possible benefits and barriers within the use in the daily clinical setting of oncology.

## Methods

### Study participants

Ten oncological clinics participated in the study. They were asked to hand out two types of questionnaires to their patients. The clinics were free to choose the clinical setting as well as the time the patients would receive the questionnaires, meaning the stage of treatment and time during a hospital stay. The patients were either ambulant or in-patient and had different stages of cancer. All patient data was anonymously taken.

### Survey

We used two questionnaires. The first questionnaire was developed by the German Program for Guidelines in Oncology and was published with the S3 Guideline Complementary Medicine in the Treatment of Oncological Patients. It contains a table listing 38 CAM procedures and a legend with three colored symbols in red, yellow, and green. The symbols red and yellow suggest talking to a physician about a certain use. Green means there are no interactions with cancer treatments known. The participants had to tick procedures that they were using. There are nine red and fifteen yellow labelled items.

The second questionnaire was developed for this study to examine the practicability of the guideline questionnaire and the communication with an HCP regarding CAM. It started with an introduction that explained the intention of the survey and the fact that participation would be anonymous, voluntary and take no more than 15 min. Written informed consent was given by filling in the questionnaire. The questionnaire consisted of 21 questions regarding the following topics:

1. Demographic data, including age, gender, education, type and time of cancer diagnosis.
2. Past use of and experience with CAM.
3. Source of information, prior consultation with an HCP.
4. Changes in interest and use of CAM after filling out the questionnaire of the S3 guideline.
5. Rise of questions, source for answers/information after filling out the questionnaire of the S3 guideline.
6. Consultation with a clinician, interest in further information.

The questionnaire included different types of questions, such as closed, open, and multiple choice.

Furthermore, we held two types of semi-structured interviews with physicians and nurses. The first type was a short interview of five minutes in the early stages of the study, which concentrated on possible barriers to an implementation of the questionnaire. The second interview of fifteen minutes and more at the end of a clinic’s survey covered the HCP’s general past experiences with CAM and patients using CAM, as well as organizational, functional, and communicational aspects of the work with the questionnaire.

### Statistical analysis

We utilized IBM SPSS Statistics 28 for the data analysis. To explore associations between variables we used three types of correlation coefficients depending on the scale levels. For correlating metric scale levels, such as age, years since diagnosis and number of CAM methods, with nominal scale levels, which includes all questions of the S3 and supporting questionnaire we used Eta squared coefficient (*η*^2^). Effect sizes of 0.01 and more were considered low, 0.06 and more medium, and 0.14 and more were considered high. Additionally, *p* values were calculated and values smaller than 0.05 were considered significant. For metric and ordinal scale levels, like level of education we used Spearman correlation. For correlating all lower scale levels, we used a chi-squared test and calculated *p* values.

### Ethics vote

The study was approved by the ethics committee from the University Clinic Jena.

## Results

### Demographic data (Table 1)

**Table 1:** Demographic data (*N* = 258)

Category	Answer	*N* (%)
Age	< 30 years	3 (1.2)
	30–50 years	38 (14.7)
	51–70 years	148 (57.4)
	71–80 years	39 (15.1)
	> 80 years	8 (3.1)
	No answer	22 (8.5)
Gender	Male	91 (35.3)
	Female	164 (63.5)
	No answer	3 (1.2)
Supporting questionnaire (*N* = 116)		
Age	< 30 years	1 (0.86)
	30–50 years	17 (14.66)
	51–70 years	77 (66.38)
	71–80 years	19 (16.38)
	> 80 years	1 (0.86)
	No answer	1 (0.86)
Gender	Male	34 (29.3)
	Female	81 (69.8)
	No answer	1 (0.9)
Education	No qualification	2 (1.7)
	Secondary school qualification	45 (38.8)
	University entrance diploma	17 (14.7)
	Vocational training	12 (10.3)
	University diploma	26 (22.4)
	No answer	14 (12.1)
Type of cancer (*N* = 116, multiple answers)	Breast cancer	33 (28.4)
	Other gynaecological cancer	5 (4.3)
	Urogenital cancer	4 (3.4)
	Colorectal cancer	6 (5.2)
	Other gastrointestinal cancer	6 (5.2)
	Lung cancer	5 (4.3)
	Leukemia/lymphoma	42 (36.2)
	Other	5 (4.3)
	No answer	13 (11.2)
Years since diagnosis	< 1 year	39 (33.6)
	1–2 years	30 (25.9)
	> 2–5 years	17 (14.6)
	> 5–10 years	10 (8.6)
	> 10 years	9 (7.8)
	No answer	11 (9.5)

In total, 258 patients filled out the questionnaire of the S3 guideline. A third of the participants were male (35.3%) and two-thirds were female (63.5%). The peak age ranged between 51 and 70 years (57.4%) with a mean of 61. Slightly more participants were older than 70 (18.2%), compared to those younger than 50 (15.9%).

116 participants additionally filled out the second questionnaire, that covers past experiences with CAM regarding use, sources, and medical consultations, as well as aspects of the experience with the S3 questionnaire. More than two third of the participants were female (69.8%), and 29.3% were male. The data on age is similar to the S3 questionnaire as the peak age ranged between 51 and 70 years (66.38%) with a mean of 60.7. The majority of participants had a secondary school qualification (38.8%) or a university diploma (22.4%). The leading cancer type was leukemia/lymphoma (36.2%), followed by breast cancer (28.4%). Most patients had been diagnosed less than a year ago (33.6%) up to 2 years prior (25.9%).

### Usage behavior prior to the survey

#### Disclosure and sources

Over 70% of 116 participants never actively dealt with CAM before filling out the questionnaire nor discussed CAM with their physician. Age shows a medium association with the fact of having dealt with CAM, the older the patients, the lesser it was likely they had dealt with it (*η*^2^ = 0.061, *p* = 0.008). 95 participants (81.9%) used some type of CAM in the past, and 54 out of 77 revealed they never disclosed their usage to a physician. A high education correlates with prior CAM use (*p* = 0.011). Of 103 participants only 46.6% knew that CAM could cause side effects and interactions. We found a weak association between knowledge about potential interactions and side effects and years since diagnosis (*η*^2^ = 0.047, *p* = 0.034).

**Fig. 1:**
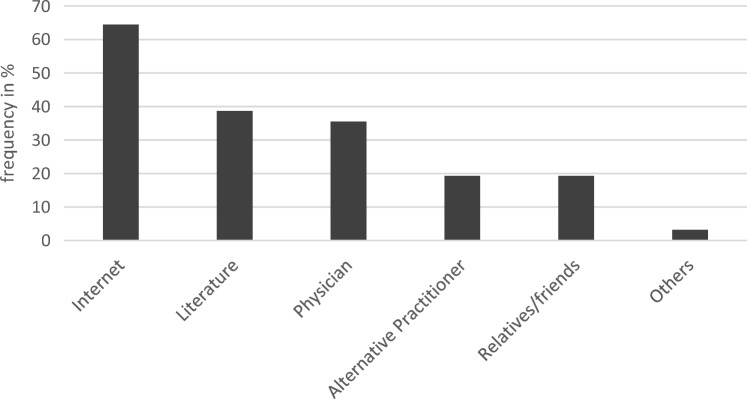
Sources on CAM in the past (*N* = 31; multiple answers possible)

31 participants elaborated on their most common sources for information on CAM (Fig. 1), the internet leading with 64.5%, followed by literature (38.7%), physicians (35.5%), alternative practitioners and relatives/friends (both 19.3%). Age and sources of information on CAM reveal a medium association with alternative practitioners correlating positively (*η*^2^ = 0.137, *p* = 0.031), and a strong with the internet correlating negatively (*η*^2^ = 0.153, *p* = 0.022). Other sources, knowledge about potential risks nor different aspects of communicating with an HCP do not show any association with age or with education. There are no associations between years since diagnosis and usage behavior or different sources of CAM in the past.

#### Overview of CAM use

**Fig. 2:**
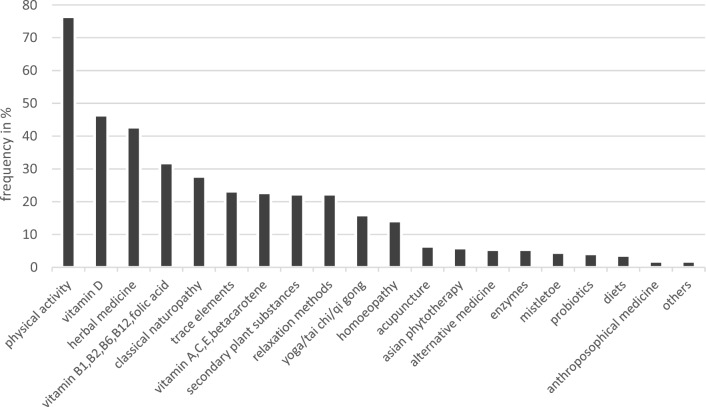
Usage of CAM methods (*N* = 220; multiple answers possible)

Out of 65 participants, 50% described their overall experience with CAM as positive, the other half as neutral, none as negative. A total of 85% used at least one of the listed CAM methods of the questionnaire. Figure 2 shows the use of these CAM methods.

The most frequently used CAM method by far was physical activity (76.4%), followed by vitamin D (46.4%) and herbal medicine (42.7%), which summarizes the methods phytotherapeutics, sage, camomile, lavender, and aroma therapy. Over 42.7% of the participants using herbal medicine combined at least two substances of this category. Vitamin D and the B-Vitamins are the only substances that are associated with demographic data, as age is correlating positively with a weak effect strength (*η*^2^ = 0.033, *p* = 0.005 and *η*^2^ = 0.042, *p* = 0.002). The physician as a source for CAM is associated with relaxation methods (*p* = 0.007) and classical naturopathy (*p* = 0.010).

The total number of reported CAM shows various medium to high associations with answers to the supporting questionnaire. To name the most relevant, we found a high association with having dealt with CAM in the past (*η*^2^ = 0.292, *p* = < 0.001) and with a positive experience with CAM in general (*η*^2^ = 0.367, *p* = < 0.001). The number of CAM also correlates positively with the number of red labeled CAM (*r*_s_ = 0.632, *p* = < 0.001). There are no associations between the number of used CAM and a specific source for CAM prior to the survey.

#### Concerning substances

**Fig. 3:**
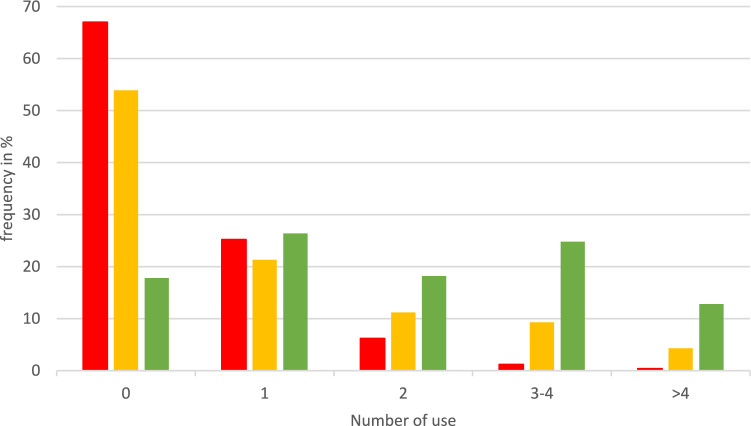
Usage of CAM by color coding (*N* = 258; multiple answers possible). Red, yellow: consultation with HCP recommended; green: no interactions with cancer drugs known

Figure 3 shows the use of CAM methods in relation to their potential interactions and side effects illustrated by color coding.

25.2% of participants used one of the red-labeled methods. A total of 7.8% used two or more red-labeled methods. Adding the usage rate of yellow-labeled CAM 16.2% used at least two of the listed CAM, which should entail consultation with an HCP. The number of red-labeled CAM reported shows a high association with seminars as a source of information on CAM prior to the survey (*η*^2^ = 0.199, *p* = 0.011), and CAM use being experienced positively (*η*^2^ = 0.249, *p* = < 0.001). It shows a medium association with having dealt with CAM in the past (*η*^2^ = 0.115, *p* = < 0.001) and having addressed CAM use to a physician (*η*^2^ = 0.123, *p* = 0.003), while awareness of potential risks is only associated with a weak effect size (*η*^2^ = 0.052, *p* = 0.026).

#### Safer substances

Crossing CAM methods of the S3 questionnaire with knowledge of potential interactions and side effects as answered in the supporting questionnaire, it is noticeable that more safer substances are associated, such as Vitamin D (*p* = 0.003), zinc (*p* = 0.033), homeopathy (*p* = 0.014), sport and exercise (*p* = 0.036) and relaxation methods (*p* = 0.041).

Education correlates with present CAM use in general (*p* = 0.012) but is not associated with any specific CAM method of the S3 questionnaire. However, we found a weak correlation between education and the number of green-labeled methods reported, which could suggest higher educated patients use CAM in a safer way (*r*_s_ = 0.232, *p* = 0.025). This stands in a slight contradiction to the unproven/lacking knowledge of risks.

### After the survey

#### Interest and questions

Of 114 participants 78.9% stated that they used one or more of the listed items of the questionnaire. 60% gained more interest in CAM after the questionnaire and it shows a medium association with the number of red-labeled CAM (*η*^2^ = 0.060, *p* = 0.019) and years since diagnosis (*η*^2^ = 0.074, *p* = 0.009), with more time since the diagnosis passing, the less interest they were gaining, one exception being the group of freshly diagnosed of under half a year. This group of patients seems less interested, which is consistent with statements made in our interviews with the HCPs. Of 102 patients 37.3% had questions during the survey and 34.1% of 85 participants left still having open questions.

#### Sources

**Fig. 4:**
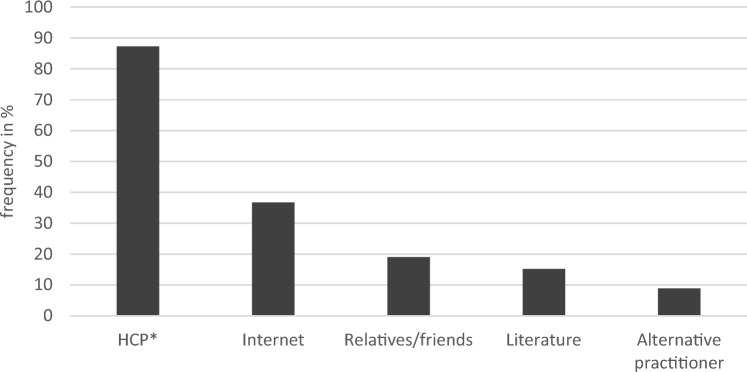
Sources for information regarding the survey (*N* = 79; multiple answers possible). *HCP = physician 73.4%, nurse 13.9%

Figure 4 shows the frequency of sources for information regarding the survey.

Out of 79 participants, the leading source to find answers to questions regarding the survey was an HCP (87.3%). Other common sources were again the internet (36.7%), followed by relatives and friends (19%), literature (15.2%) and alternative practitioners (8.9%) Out of 57 patients, who consulted an HCP 77.2% saw their questions appropriately answered and 80.2% felt that there was enough time for the conversation. The total number of CAM and the number of red-labeled CAM are highly associated with asking an alternative practitioner about questions that arose in the survey (*η*^2^ = 0.284, *p* = < 0.001; *η*^2^ = 0.249, *p* = < 0.001). We have not found associations between years since diagnosis and sources for questions during the survey nor any perceptions on consulting an HCP. Age is not associated with feelings about an HCP consultation as well, while gender is not associated with any usage behavior, that was covered in the questionnaire.

#### Effect of the legend

Because the clinics were free to either leave or remove the legend, from 116 questionnaires only 59 contained the symbols. For two third of these participants, the legend was educational, 10% felt unsettled by the red and yellow labeling.

#### Desire for consultation

About one-third of the 258 participants filling out the S3 questionnaire had a desire for consultation or questions about CAM, two third either did not want a consultation or did not give an answer. This result fits with the answers in the supporting questionnaire, as 37.9% out of 95 patients stated that they were interested in further information on CAM in the form of online lectures.

Interest in an online lecture is highly associated with the total number of reported CAM (*η*^2^ = 0.157, *p* = < 0.001), and shows medium association with the number of red-labeled CAM (*η*^2^ = 0.095, *p* = 0.004). Appropriately for the correlation between age and using the internet as a source on CAM, interest in online lectures correlates negatively with age as well (*η*^2^ = 0.092, *p* = 0.003).

#### Associations between past usage behavior and survey experience/effect

Having dealt with CAM and talked to a physician about CAM in the past show similar associations, as they both correlate with the knowledge of potential interactions and side effects (*p* = < 0.001 and *p* = 0.002), a higher interest in CAM (*p* = 0.007 and *p* = 0.018) and in online lectures for further information (*p* = < 0.001 and *p* = 0.005) after participating in the survey. Both features are associated with seeking answers within an HCP (each *p* = 0.016) and in literature (*p* = 0.019 and *p* = 0.002) when questions through the survey arose. Those who had dealt with CAM in the past had a change in their usage behavior (*p* = 0.008) and were more likely to seek answers from an alternative practitioner (*p* = 0.018). It is not associated with the perception of the quality or time regarding the HCP consultation nor any remaining open questions.

An alternative practitioner as a source of information correlates with a changed usage behavior (*p* = < 0.001) and questions while filling out the questionnaire (*p* = 0.007). It is not associated with knowledge of risks within CAM use.

The total number of reported CAM is highly associated with a changed usage behavior after completing the survey (*η*^2^ = 0.154, *p* = < 0.001). General CAM use in the past also correlates with a changed usage behavior (*p* = 0.020), as well as knowledge about interactions and side effects (*p* = 0.013), and an interest in CAM through the survey (*p* = 0.002). It is also associated with interest in an online lecture for further education on CAM (*p* = 0.022) and still having open questions (*p* = 0.020). We haven’t found any associations between prior CAM use and specific sources while trying to find answers or how patients perceived a consultation with an HCP.

We found positive correlations between good experience with CAM use and interest in CAM through the survey (*p* = 0.002), a changed usage behavior (*p* = 0.005) and trying to find answers to topics of the questionnaire through an alternative practitioner (*p* = 0.008), whereas there are no associations with present CAM use in general, knowledge of risks, any other sources on the information including a consultation with an HCP or remaining questions.

### Short interviews

After 2 months into the study, we conducted short interviews with nurses and one secretary of the chief of medicine from three medical institutions participating in the study, together with nurses from three other institutions who were not participating. The aim of these interviews was to find out barriers to the implementation of the questionnaire. Four out of six stated organizational potential barriers such as internal authorisation and preparatory training in the field of CAM. Two interview partners participating in the study and one that was not cited, that patients needed help in filling out the survey. Moreover, the consequent need for further discussion on CAM raises concerns due to a lack of competence with respect to CAM on the side of the physicians as well as a shortage of suitable staff in general. Furthermore, even though partly a large number of questionnaires was handed out, the response rate was distinctly lower. Two out of six interview partners explained patients seemed weary of surveys, but the ultimate cause could not be stated with certainty.

### Final interviews

At the end of the surveys, we held interviews with six clinicians. The interview partners were those, who also conducted the survey in their clinic. Three participants were nurses with 4–20 years of experience and three were physicians with 5–25 years of experience. One nurse questioned only in-patients, one physician only out-patients. The others used the questionnaire in both settings, without feeling that one setting was significantly more favorable than the other one.

#### Prior knowledge and experience with CAM

The interviewees learned about CAM during their oncological work and through further education. In addition, four out of six had a certificate for integrative oncology from the workgroup PRiO (engl: Prevention and Integrative Oncology) of the German Cancer Society. In their daily work, the main sources for questions regarding CAM were the S3 Guideline Complementary and Alternative Medicine (66.6%), the information on the German website of “Stiftung Perspektiven” (engl: Foundation Perspectives) and literature (each 33.3%).

Five interview partners experienced CAM patients to be very proactive in matters of their health and well-informed about CAM. They also described the typical clientele to be more educated and from a higher social class. Two out of six said they were typically younger than 75 years, and that present family members often show high interest in CAM to support their relative’s recovery and health. One nurse stated if being asked about CAM directly, patients give out a lot of information on their usage, but they often do not realize what substances classify as CAM. Patients with no interest in CAM were often known to be from low social and educated classes (83.3%), older (above 75 years) (33.3%), and from foreign countries (33.3%).

#### Barriers

50% of the interviewees highlighted that they specifically selected the patients, they would give the questionnaire. They reasoned that they could assess, which patient would be interested in discussing CAM or possibly already used some type of CAM. It was also an issue of time and effort because the HCPs feared it would take too much time to explain the whole concept of CAM to patients, who never dealt with it before. Four out of six experienced an increased workload because they were solely responsible for distributing and collecting the questionnaires, and for handling the patients need for talks.

Three out of six stated another issue to be the use of the questionnaire at an inconvenient time during the hospital stay or course of the disease, such as right before or after operation, at the time of admission, and shortly after a diagnosis. In two clinics the HCPs noticed that in-patients were reluctant to fill out the questionnaires. They were overwhelmed with the number of daily tasks in the ward as is, as well as weary of questionnaires in general. Two interview partners explained that some patients like foreign and old ones had trouble filling out the questionnaire because they were deterred by the number of items and did not understand or know the listed methods. Especially with these patients reassuring and affirming communication was necessary to take away the first overwhelming feeling. Another problem that one oncological nurse stated was mixed preparations. In some cases, the sheer number of ingredients, some of which were not covered by the questionnaire, made it impossible to make evidence-based statements about the potential of interactions.

#### Positives

With four out of six interview partners one of the most mentioned positive aspects of the questionnaire was the initiation of further conversation. With the variety of items, it covered a broad spectrum of methods that a standard medical history usually would not cover. All HCPs described that patients were either neutral to open, or as three HCPs explained even thankful and happy to be able to talk about CAM and to get a nudge to read more on unknown substances. Two nurses strongly emphasized that the questionnaire created a focus on CAM and patients felt taken seriously. One nurse, that used the questionnaire solely in consultations for patients going into chemotherapy stated it increased safety within CAM usage because critical substances were discovered in a standardized way and patients were made aware of interactive potentials and possibly harming substances.

#### Need for talk

The most common questions in general were specifics about certain substances, that were either used and labeled red or yellow, unknown, or not listed. For the most part all interviewees characterized the capacity of those conversations as appropriate. However, one oncologist experienced that about 20% of 20 patients required one-hour-long conversations about CAM after participating in the survey, that forced her to do overtime.

#### The legend

As already mentioned, the clinics were free to leave or remove the legend in the form of colored symbols at the side of the list. Our interviewees left it, so everyone was able to report on their experiences. Two HCPs didn’t find patients to react to the legend, describing the effect as neutral, though one copied the questionnaires only in black and white. Three said the legend was helpful and educational for patients and as two stated also for themselves, because it functioned as a main thread for the conversation. One physician had a different experience. She suspected patients to be unsettled and influenced by the legend and not be entirely honest about certain usages that may be labeled red. One other nurse mentioned similar incidents, in which she strongly emphasized the risk of using certain methods while undergoing active treatments.

## Discussion

The aim of this study was to investigate the practicability of the S3 questionnaire and to assess its positive effects and barriers for establishing a standardized way to cover CAM usage in daily oncological work. We conducted interviews with HCPs and evaluated patient questionnaires on CAM usage and communicational behaviors.

### Prevalence

More than 80% of the participants have used some type of CAM in the past, and similarly many used at least one of the listed CAM methods at the time of questioning, which exceeds other results of recent studies that had a usage rate that ranged from 36 to 60% (Lederer et al. 2021; Wode et al. 2019; Ciarlo et al. 2021). However, Davis, Oh, Butow et al. described the prevalence of CAM widely ranging from 11 to 95% (Davis et al. 2012) aligning with another systematic literature review by Alsharif, that found a prevalence of 25–80% (Alsharif 2021), which brings our results back in line with scientific data, but still at the upper end. The inclusion of different methods in the wide field of CAM may explain the heterogenous rates found in studies using different questionnaires (Horneber et al. 2012). Physical activity, phrased sports and movement was the leading CAM method with around three quarters. Even by factoring out this very common method, the prevalence of CAM use is still rather high at 74%. In another contrast to the results of other researchers, there was no association between gender and usage behavior. Reason could be that some clinicians have weighed which patients to question. This circumstance may have resulted in a one-sided survey group/caused bias. CAM use and dealing with CAM were associated with young age and high education, which is consistent with other study findings (Alsharif 2021; Wode et al. 2019; Keene et al. 2019) and the experiences made by the majority of our interviewed HCPs.

### Critical usage behavior

Around one-quarter of 258 participants used one of the nine listed red-labeled CAM and one in six used at least two red or yellow-labeled CAM, that suggest consulting with an HCP. The more methods a patient used, the riskier substances, meaning red-labeled items they used, too. This usage behavior is viewed critical as evidence shows a higher risk of potential interactions and side effects (Leitlinienprogramm Onkologie (Deutsche Krebsgesellschaft 2021). In addition, half of the participants never disclosed their usage to a physician and the same amount did not know of CAM’s potential to cause side effects and interactions. This highlights the necessity of covering the medical history on CAM and the need for educating patients and it points to the importance of the recommendation of the guideline to use a structured way to assess CAM usage. Patients using multiple methods and substances had a very positive, open, and interested view of CAM. They reported to visit alternative practitioners and seminars on complementary and alternative topics. But most often they were not aware of potential side effects and interactions. This suggests insufficient clarification by these sources, even though interactions between complementary substances and medication for cancer treatment and other comorbidities are very common. Prevalence of potential or occurred interaction ranges from 30 to 90% with different sample groups (Loquai et al. 2016; Prely et al. 2022; Agbabiaka et al. 2018). Interaction with anticancer drugs specifically varies between 40 and 65% (Prely et al. 2022). On the other hand, the report of physicians as a source on CAM goes along with the knowledge of interactions and side effects, suggesting that physicians inform more about the potential risks of CAM use. By assessing CAM use by default HCPs can become aware of any critical usage behavior at an early stage and then discuss it with these patients. As part of self-efficacy and self-responsibility patients are also able to inform themselves on these matters, as the ones who have dealt with CAM knew more often about the potential interactions and side effects. Ultimately, when patients did know of the potential of side effects and interactions, they used more safe substances, which hints that by educating patients on CAM, they are able to opt for low-risk methods. Specifically, the methods of relaxation and classical naturopathy were associated with physicians as a source for CAM. In line with these results, the systematic review of Davis, Oh, Butow et al. stated that one study had found the highest rates of disclosure for naturopathy (Davis et al. 2012). There was a weak correlation between the level of education and using green-labeled CAM. However, this is only a hint that highly educated people might use CAM in a more differentiated way, and there was no significant association with knowledge of interactions and side effects. In fact, we haven’t found proof for any sociodemographic factor predicting safe CAM use.

### Benefits in using a standardized questionnaire

The percentage of patients talking to an HCP regarding CAM increased significantly from about thirty-five percent to nearly ninety percent after the survey and emphasized the effect of using a standardized questionnaire. By increasing inquiry and displaying interest and the willingness to listen, the participating centers increased the rate of disclosure (Davis et al. 2012). This is consistent with the interviewees professional experiences that patients already disclose a lot about their usage when being asked about it. They also stated that the questionnaire initiated further conversations with patients. Using a standardized questionnaire opens a dialogue on CAM and broadens the knowledge of patients’ medical history, which otherwise could have been missed or be incomplete (Balneaves and Watling 2022). Furthermore, the interviewed HCPs explained that patients were thankful to talk about their CAM interest and usage. More importantly, they felt taken seriously. In regard, many studies showed that including CAM in the conversation with a cancer patient improves the relationship between HCP and patient and enhances patient-centeredness (Tilburt et al. 2019; Stie et al. 2020; Rogge et al. 2021).

Patients with a more recent diagnosis had gained more interest in CAM through the survey compared to patients with a longer-standing diagnosis, which could be related to patients with a more recent diagnosis being of younger age. Patients using several CAM methods were more likely to change their usage behavior after the survey. In the questionnaire, we have not specified the change in usage behavior further, so it could mean anything from dealing with information on CAM in a different way or developing an awareness or literally changing the use of a certain method. Nevertheless, it’s an indication of an aimed effect of the survey, though we do not know for certain how well a consultation with an HCP was perceived and its impact on the reported change.

The internet remained one of the most frequent sources after participating in the survey. Especially younger patients more often consulted the internet on CAM, lining up with recent data about information sources for CAM (Ciarlo et al. 2021). With non-disclosure being very common (Akeeb et al. 2022; Lederer et al. 2021) and patients using sources such as the internet or seminars frequently, the question rises of how well information about scientific evidence and potential risks is provided by these sources. A study on the quality of information on drug-CAM interactions on the internet concluded a poor clarification (Scarton et al. 2013). Another study that evaluated the quality of adult education courses on CAM in Germany concluded that many courses are instructed by non-medicals without proper training in scientific evaluation, who advertise alternative and non-evidence-based subjects (Ott et al. 2022). In this regard it is important that HCPs share evidence-based information, e.g. from guidelines such as the S3 Guideline so that patients are effectively counseled on their use of CAM (Latte-Naor and Mao 2019), learn to use other sources and also regard methods or substances offered in a free market in a more educated way (Scarton et al. 2013).

### Challenges with structured assessment of CAM use

Institutions lack the implementation of standard procedures and resources that help CCPs discuss CAM on a regular basis (Balneaves and Watling 2022). In this regard, in the first short interviews a concern due to a shortage of suitable staff was addressed, as well as a lack of competence with respect to CAM, which also echoed in the final interviews. In fact, the participating centers mostly had at least one physician with some professional education in CAM. Mostly this person or the nurses working in his/her team distributed the questionnaires. In consequence, there was only a limited amount of time available for discussions on CAM and the interviewees reported that a generalized distribution to all patients would not be feasible in routine care. As a consequence, patients that use CAM or would be interested in consultation could be overlooked, as a main reason for non-disclosure is non-inquiry (Davis et al. 2012; Wode et al. 2019), indicating the necessity to actively ask patients about their CAM usage. Studies suggest that patients are more likely to discuss their use of CAM when their HCP expects them to use some form of CAM (Davis et al. 2012). In clinics with a referral system for CAM patients, the use of the questionnaire was perceived more practicable for day-to-day work beyond the study. In case a reaction to the filled-in questionnaire is not possible due to lack of time or knowledge it poses an ethical problem, which in fact is not solved by cancelling a screening. Even though most interviewed HCPs stated the capacity of following conversations as appropriate, the chronic shortage of time in clinical work is a major hurdle for many HCPs to assess CAM usage regularly (Balneaves and Watling 2022). Conversations including CAM are usually longer than the ones that do not address CAM, but at the same time also more patient-centered (Tilburt et al. 2019), showing the controversy of patient needs and the struggle to meet those needs. Patients that used red-labeled substances continued to do so in the past, even though they had addressed CAM use to a physician, suggesting that the communication between patient and doctor had not been sufficient. Non-compliance could be due to patients seeking to maintain control over their treatment or the doctor’s inability to provide information on CAM (Davis et al. 2012). The existing continuing need for improving education on CAM should start with medical students, including nurses and CAM updates should be integrated for example as part of seminars or workshops on updates of relevant guidelines.

### Pros and cons on utilizing the legend

As for the patients, two third felt the legend was educational, which two of the interviewed six HCPs confirmed when asked, and one independently. Some also stated the legend was helpful and educational for themselves because they could focus better on critical issues in follow-ups with patients, which was seen as a strong benefit. On the other hand, 10% of the patients felt unsettled by the red and yellow labeling, which might make them under-reporting critical usage especially in case of lacking trust in the physician and his expertise on the topic. Two interviewees mentioned such thoughts, as they suspected some patients to hide certain usages from them, which could be grounded in the anticipation of the doctor’s disapproval or negative attitude (Wode et al. 2019; Lederer et al. 2021; Davis et al. 2012). This emphasizes the need for effective and non-judgmental communication on potential risks of using CAM methods. Using the legend may also result in more frequent and longer consultations, as questions from patients focused on substances labeled red or yellow. Therefore, a few clinics decided to remove the legend beforehand. In sum, the positive opinions about the legend predominated.

## Limitations

There are a few limitations to our study that should be considered. First of all, only centers open-minded and already offering some counseling on CAM participated in the study. Our demographic data shows that our collective is not representative as there is a high rate of female and highly educated persons. In part, this may be due to some clinicians preselecting patients whom they thought to be interested in CAM or already using CAM. In addition, the number of interviews is rather small, due to a lack of physicians or nurses from participating centers having time for them. On the other hand, following a grounding theory concept, we saw that in the last two interviews no new aspects were collected (Moura et al. 2021).

## Conclusion

CAM is highly important in the work with cancer patients. In our study the prevalence of CAM use was high, as well as rates of non-disclosure and insufficient knowledge of potential risks within CAM use. Especially patients who use many methods and especially substances must be met in their interest by HCPs to ensure safe usage. Our study highlights in many ways the necessity of covering the medical history of CAM and the need for educating patients, which supports the recommendation of the guideline to use a structured way to assess CAM usage. A standardized questionnaire may increase the rate of disclosure and induce safer usage behavior. It may open a dialogue on CAM and improve the relationships with patients as they feel being taken seriously. A helpful tool in the questionnaire is the legend that described potentials of methods to cause interactions and side effects by color coding. It may inform patients and support HCPs in their work with the questionnaire but needs effective communication on the reason behind the color coding. The chances of using a standardized questionnaire for CAM use are clear. Barriers to implementation are grounded in the continuing need for improved education and the chronic lack of time within clinical work. Institutions should focus on implementing standard procedures and resources that help CCPs discuss CAM on a regular basis. CCPs in turn must meet the patient’s demands for CAM counseling and make sure they are equipped professionally.

## References

[CR1] Agbabiaka TB, Spencer NH, Khanom S, Goodman C (2018). Prevalence of drug-herb and drug-supplement interactions in older adults: a cross-sectional survey. Br J Gen Pract.

[CR2] Akeeb AA, King SM, Olaku O, White JD (2022). Communication between cancer patients and physicians about complementary and alternative medicine: a systematic review. J Integr Complement Med.

[CR3] Alsharif F (2021). Discovering the use of complementary and alternative medicine in oncology patients: a systematic literature review. Evid Based Complement Alternat Med.

[CR4] Balneaves LG, Watling CZ (2022). "Part of the Conversation": a qualitative study of oncology healthcare professionals' experiences of integrating standardized assessment and documentation of complementary medicine. Integr Cancer Ther.

[CR5] Beyer M, Scherer M, Wollny A, Chenot JF, Baum E, Gerlach FM (2010). Redesigning the guideline development concept (“Zehnstufenplan”) of the german college of general practitioners and family physicians. Z Allg Med.

[CR6] Ciarlo G, Ahmadi E, Welter S, Hubner J (2021). Factors influencing the usage of complementary and alternative medicine by patients with cancer. Complement Ther Clin Pract.

[CR7] Conrad AC, Muenstedt K, Micke O, Prott FJ, Muecke R, Huebner J (2014). Attitudes of members of the German Society for Palliative Medicine toward complementary and alternative medicine for cancer patients. J Cancer Res Clin Oncol.

[CR8] Cramer H, Cohen L, Dobos G, Witt CM (2013). Integrative oncology: best of both worlds-theoretical, practical, and research issues. Evid Based Complement Alternat Med.

[CR9] Davis EL, Oh B, Butow PN, Mullan BA, Clarke S (2012). Cancer patient disclosure and patient-doctor communication of complementary and alternative medicine use: a systematic review. Oncologist.

[CR10] Deutsche Krebsgesellschaft e.V. n.d. Onkologische Leitlinien im Überblick. Accessed 13 Jan. https://www.krebsgesellschaft.de/deutsche-krebsgesellschaft-wtrl/deutsche-krebsgesellschaft/leitlinien/onkologische-leitlinien-im-ueberblick.html

[CR11] Gras M, Vallard A, Brosse C, Beneton A, Sotton S, Guyotat D, Fournel P, Daguenet E, Magné N, Morisson S (2019). Use of complementary and alternative medicines among cancer patients: a single-center study. Oncology.

[CR12] Horneber M, Bueschel G, Dennert G, Less D, Ritter E, Zwahlen M (2012). How many cancer patients use complementary and alternative medicine: a systematic review and metaanalysis. Integr Cancer Ther.

[CR13] Keene MR, Heslop IM, Sabesan SS, Glass BD (2019). Complementary and alternative medicine use in cancer: a systematic review. Complement Ther Clin Pract.

[CR14] Klein GE, Guethlin C (2018). Information and training needs regarding complementary and alternative medicine: a cross-sectional study of cancer care providers in Germany. Integr Cancer Ther.

[CR15] Latte-Naor S, Mao JJ (2019). Putting integrative oncology into practice: concepts and approaches. J Oncol Pract.

[CR16] Lederer AK, Baginski A, Raab L, Joos S, Valentini J, Klocke C, Samstag Y, Hübner K, Andreeva I, Simmet T, Syrovets T, Hafner S, Freisinger A, Storz MA, Huber R (2021). Complementary medicine in Germany: a multi-centre cross-sectional survey on the usage by and the needs of patients hospitalized in university medical centers. BMC Complement Med Ther.

[CR17] Leitlinienprogramm Onkologie (Deutsche Krebsgesellschaft, Deutsche Krebshilfe, AWMF) (2021) Komplementärmedizin in der Behandlung von onkologischen PatientInnen, Langversion 1.1 (AWMF Registernummer: 032/055OL).

[CR18] Loquai C, Dechent D, Garzarolli M, Kaatz M, Kaehler KC, Kurschat P, Meiss F, Stein A, Nashan D, Micke O, Muecke R, Muenstedt K, Stoll C, Schmidtmann I, Huebner J (2016). Risk of interactions between complementary and alternative medicine and medication for comorbidities in patients with melanoma. Med Oncol.

[CR19] Moura CO, Silva ÍR, Silva TPD, Santos KA, Crespo M, Silva MMD (2021). Methodological path to reach the degree of saturation in qualitative research: grounded theory. Rev Bras Enferm.

[CR20] Ott KU, Keinki C, Kaesmann L, Huebner J (2022) Education of complementary and alternative medicine in adult education centers in Germany: a web-based survey. Wien Med Wochenschr 10.1007/s10354-022-00951-0 PMC11130027 35948702

[CR21] Prely H, Herledan C, Caffin AG, Baudouin A, Larbre V, Maire M, Schwiertz V, Vantard N, Ranchon F, Rioufol C (2022). Real-life drug-drug and herb-drug interactions in outpatients taking oral anticancer drugs: comparison with databases. J Cancer Res Clin Oncol.

[CR22] Rogge AA, Helmer SM, King R, Canella C, Icke K, Pach D, Witt CM (2021). Effects of training oncology physicians advising patients on complementary and integrative therapies on patient-reported outcomes: a multicenter, cluster-randomized trial. Cancer.

[CR23] Scarton LA, Del Fiol G, Treitler-Zeng Q (2013). Completeness, accuracy, and presentation of information on interactions between prescription drugs and alternative medicines: an internet review. Stud Health Technol Inform.

[CR24] Stie M, Jensen LH, Delmar C, Nørgaard B (2020). Open dialogue about complementary and alternative medicine (CAM) integrated in conventional oncology care, characteristics and impact. A systematic review. Patient Educ Couns.

[CR25] The National Center for Complementary and Integrative Health (2021) Complementary, alternative, or integrative health: what’s in a name? https://www.nccih.nih.gov/health/complementary-alternative-or-integrative-health-whats-in-a-name. Accessed 28 Dec

[CR26] Tilburt J, Yost KJ, Lenz HJ, Zúñiga ML, O'Byrne T, Branda ME, Leppin AL, Kimball B, Fernandez C, Jatoi A, Barwise A, Kumbamu A, Montori V, Koenig BA, Geller G, Larson S, Roter DL (2019). A multicenter comparison of complementary and alternative medicine (CAM) discussions in oncology care: the role of time, patient-centeredness, and practice context. Oncologist.

[CR27] Trimborn A, Senf B, Muenstedt K, Buentzel J, Micke O, Muecke R, Prott FJ, Wicker S, Huebner J (2013). Attitude of employees of a university clinic to complementary and alternative medicine in oncology. Ann Oncol.

[CR28] Wode K, Henriksson R, Sharp L, Stoltenberg A, Hök Nordberg J (2019). Cancer patients' use of complementary and alternative medicine in Sweden: a cross-sectional study. BMC Complement Altern Med.

[CR29] Wortmann JK, Bremer A, Eich HT, Wortmann HP, Schuster A, Fühner J, Büntzel J, Muecke R, Prott FJ, Huebner J (2016). Use of complementary and alternative medicine by patients with cancer: a cross-sectional study at different points of cancer care. Med Oncol.

